# Geographical Differences and Temporal Dynamics of Intestinal Microbiota in Endangered Great Bustard *Otis tarda* Based on Environmental DNA Metabarcoding

**DOI:** 10.1155/ijm/5587641

**Published:** 2025-12-03

**Authors:** Feimin Yuan, Yiqun Wu, Guan Liu

**Affiliations:** ^1^College of Environmental and Life Science, Weinan Normal University, Weinan, Shaanxi, China; ^2^Key Laboratory for Ecology and Environment of River Wetlands in Shaanxi Province, Weinan, Shaanxi, China

**Keywords:** conservation, fecal eDNA, habitat adaptation, intestinal microbiota, temporal variation

## Abstract

Intestinal microbiota plays a crucial role in host physiological adaptation, though research on the characteristics of intestinal microbiota in the endangered great bustard *Otis tarda* has been initiated, with prior studies focusing on gut microbial composition, diversity dynamics, and the impacts of captivity and overwintering periods. Comprehensive insights into geographical differences and short-term temporal dynamics across diverse habitats remain limited. Here, we used fecal environmental (eDNA) metabarcoding to investigate the geographical differences and temporal dynamics of the intestinal microbiota in great bustards from Hebei (HB), Inner Mongolia Autonomous Region (NMG), and Shaanxi (SX) provinces of China, with temporal sampling in two sites (the confluence area of the Yellow River and the Weihe and the Luohe rivers) of SX during December 2024–March 2025. Results revealed that the great bustard intestinal microbiota was dominated by Firmicutes, Proteobacteria, and Bacteroidota at the phylum level, with core genera including *Lachnoclostridium*, *Subdoligranulum*, and *Blaut*ia. Significant geographical divergence was observed in the NMG population (grassland habitat), which exhibited a unique enrichment of Verrucomicrobiota (especially *Akkermansia*), while SX (farmland) and HB populations were dominated by Firmicutes. Temporal dynamics in SX showed fluctuations in microbial diversity and composition, which may be linked to temporal dietary shifts in winter (inferred from habitat vegetation characteristics, as direct diet measurement was not conducted). Functional predictions indicated conserved metabolic functions across populations, with variations in genetic information processing and environmental adaptation-related functions. These findings highlight that the great bustard's intestinal microbiota may be shaped by habitat-specific factors (i.e., diet and environment, inferred from habitat type) and temporal changes, providing insights into putative microbial mechanisms underlying the ecological adaptation of the endangered great bustard *O. tarda*. This study contributes to understanding host–microbiota interactions in endangered avians and supports evidence-based conservation strategies.

## 1. Introduction

The intestinal microbiota constitutes a complex assemblage of microorganisms, including bacteria, archaea, fungi, and viruses, that colonize the gastrointestinal tracts of animals [1, 2]. These communities form intimate symbiotic relationships with their hosts, mediating a suite of critical physiological processes [3, 4]. Functionally, they facilitate nutrient metabolism by decomposing complex carbohydrates, lipids, and proteins inaccessible to the host's digestive enzymes, thereby supporting energy acquisition and biosynthesis of essential vitamins (e.g., B vitamins and vitamin K) [5, 6]. Beyond nutrition, the intestinal microbiota plays a pivotal role in immune modulation, promoting the development of the host's immune system, reinforcing intestinal barrier integrity, and inhibiting pathogenic colonization through competitive exclusion and secretion of antimicrobial compounds [7, 8]. Accumulating evidence further highlights their involvement in regulating host behavior, stress responses, and adaptive strategies to environmental fluctuations, positioning them as key mediators of host–environment interactions [9, 10].

Avian intestinal microbiota research remains underdeveloped relative to mammalian studies. Although research on wild and endangered species—including bustards—has advanced in recent years, there is still a notable paucity of data on these species [11–19]. For bustards specifically, prior studies have provided foundational insights. For example, researchers investigated the aerobic bacterial flora of captive houbara (*Chlamydotis undulata*), kori (*Ardeotis kori*), and rufous-crested (*Eupodotis ruficrista*) bustards, identifying core taxa such as *Escherichia coli* and *Staphylococcus aureus* and genera like *Aeromonas*, *Bacillus*, and *Salmonella* while also noting fungal colonization by *Aspergillus* and *Candida albicans* [12]. For the great bustard (*Otis tarda dybowskii*), another team conducted consecutive-year surveys of wild wintering populations and temporal dynamic analyses during overwintering, confirming that its gut microbiota is dominated by Firmicutes, Bacteroidetes, Actinobacteria, and Proteobacteria, with core genera including *Butyrivibrio*, unclassified_f_Lachnospiraceae, and *Oscillibacter*, and highlighting stable interannual microbial composition under consistent wintering habitats [13, 14]. Additionally, a separate study compared the gut microbiota of captive and wild great bustards, revealing that captivity reduces bacterial alpha diversity and shifts community structure—with Firmicutes abundance decreasing and Bacteroidota increasing in captive individuals—linked to dietary changes (e.g., high-fat and high-protein artificial feeds) and restricted environmental microbial exposure [15].

Existing avian studies more broadly have primarily focused on common or model taxa—such as passerines (*Passer domesticus*), waterfowl (*Anas platyrhynchos*), and poultry—revealing that avian gut communities are typically dominated by Firmicutes, Proteobacteria, and Bacteroidota consistent with their roles in energy metabolism and dietary adaptation [19–22]. For example, studies on the globally endangered Sichuan partridge (*Arborophila rufipectus*) demonstrated significant seasonal shifts in gut microbiota linked to dietary changes between breeding and nonbreeding seasons, highlighting the tight association between temporal microbial dynamics and host foraging strategies [19]. In migratory passerines like the blackpoll warbler (*Setophaga striata*), gut microbiota composition diverged significantly between breeding grounds (Alaska) and wintering grounds (Caribbean), driven by differences in local vegetation and insect communities [20]. Even for nonmigratory species, such as the northern bald ibis (*Geronticus eremita*), gut microbiota showed geographical variation across captive breeding centers, attributed to differences in artificial diets and environmental microbial exposure [18]. For the great bustard specifically, while prior research has clarified core microbial composition, diversity dynamics under captivity and overwintering, and interannual stability, critical gaps remain: Geographical differences in microbiota across its diverse habitats (grasslands, farmlands, and riparian areas) and short-term temporal shifts (monthly scales) during winter—key to understanding ecological adaptation—have not been systematically explored. This gap limits our understanding of how intestinal microbiota support the great bustard's survival across variable habitats, hindering evidence-based conservation efforts for this vulnerable species.

Fecal eDNA metabarcoding has emerged as a transformative tool for studying intestinal microbiota in wild animals, especially endangered species [23, 24]. Its noninvasiveness avoids disrupting sensitive populations, enabling large-scale sampling across spatial and temporal gradients [25–27]—particularly valuable for elusive species like the great bustard *O. tarda*. Nonetheless, this approach has limitations: DNA degradation in nonfresh samples can bias community profiling [28–31]. Rigorous field protocols—such as targeting freshly deposited feces, immediate low-temperature storage, and rapid laboratory processing—are therefore critical to ensuring data accuracy, as implemented in this study.

The great bustard *O. tarda* is a globally endangered bird and a nationally protected species in China [32, 33], with populations declining due to habitat loss, agricultural intensification, and climate change [34, 35]. As a large terrestrial bird with seasonal migration, it feeds mainly on plant seeds and insects—dietary components widespread among Gruiformes and numerous avian groups [32, 36], and its survival and reproduction depend heavily on physiological adaptation, a process in which intestinal microbiota likely play a key role. To date, however, while prior studies have illuminated core gut microbiota composition, captivity impacts, and overwintering temporal dynamics of the great bustard [13–15], systematic analysis of microbiota differences driven by geographic variation (across provinces with distinct habitats) and short-term temporal dynamics (monthly scales during winter) remains lacking.. This gap is consequential: Habitat differences across its range (e.g., Hebei Province [HB], Shaanxi Province [SX], and Inner Mongolia Autonomous Region [NMG] of China) may drive adaptive divergence in microbiota due to variations in climate, vegetation, and food resources—for instance, grassland diets (high in recalcitrant fiber) versus farmland diets (rich in starch) could select for distinct microbial functional groups. Meanwhile, continuous sampling in SX could reveal short-term microbial shifts linked to temporal environmental changes (e.g., monthly fluctuations in food availability and temperature), providing insights into the species' rapid adaptive responses via microbiota.

This study is aimed at addressing these critical knowledge gaps using fecal eDNA metabarcoding of great bustard populations. Specifically, we seek to (1) characterize the core taxonomic composition of the great bustard's intestinal microbiota; (2) assess how geographic factors—both across provinces (HB, SX, and NMG) and between SX sampling sites (Huazhou District and Dali County)—shape microbiota structure; (3) analyze temporal dynamics of microbiota in SX samples across 4-month intervals; and (4) explore associations between microbiota, host ecological adaptation, and environmental factors (e.g., temperature and food availability). By revealing the geographic and temporal patterns of the great bustard's intestinal microbiota, this research will enhance our understanding of microbial roles in host adaptation, providing a scientific foundation for the conservation and management of the endangered great bustard *O. tarda*—including optimizing captive breeding and rescue strategies.

## 2. Materials and Methods

### 2.1. Sample Collection

**Figure 1:**
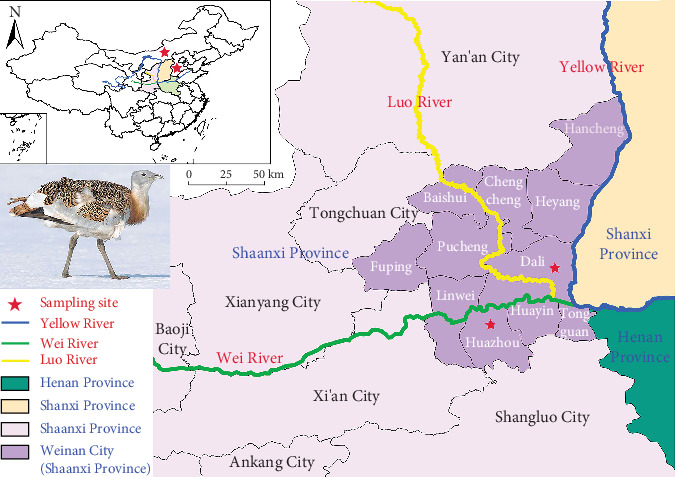
Overview of the study area, sampling sites, and distribution of geographical and ecological elements.

From December 2024 to March 2025, we conducted fecal sample collection of great bustard *O. tarda* in the habitats across SX Province, HB Province, and the NMG in China (Figure 1; Supporting Information 3: Table S1).

Habitat characterization of sampling sites: (1) HB: The sampling area is located in the southern plain of HB, characterized by a warm-temperate semihumid climate with an average winter temperature of −2°C to 5°C. Land use is dominated by irrigated farmland (mainly planting wheat, corn, and oilseed rape), interspersed with scattered artificial shelterbelts (poplar and willow). Natural vegetation is sparse, mainly consisting of winter weeds (e.g., *Capsella bursa*) in farmland gaps. (2) NMG: The sampling sites are situated in the eastern grassland of NMG, belonging to a temperate semiarid climate with harsh winter conditions (average temperature −15°C to −5°C). Land use is typical temperate steppe with no agricultural cultivation; dominant vegetation includes perennial grasses (e.g., *Stipa grandis* and *Leymus chinensis*) and forbs (e.g., *Artemisia frigida*), with low plant coverage (30%–50%) in winter. (3) SX: The two sampling sites (Huazhou District, SXHZ; Dali County, SXDL) are located in the confluence area of the Yellow River and the Weihe and Luohe rivers, with a warm-temperate continental monsoon climate (winter average temperature 1°C–3°C). Land use is a mixture of riparian farmland (wheat and cotton) and wetland vegetation (*Phragmites australis* and *Typha angustifolia*) along the riverbanks; farmland residues and aquatic plant seeds are the main food sources for overwintering great bustards.

Specifically, in the confluence area of the Yellow River and the Weihe and Luohe rivers in SX Province, two sampling sites, that is, Huazhou District (SXHZ) and Dali County (SXDL), were established along the Weihe River and the Yellow River, respectively, for synchronous collection of fresh fecal samples. All of the above samples contain at least three biological replicates. Great bustard fecal samples collected at the same location and time were derived from the same population. Each sampling site was set with three biological replicates, which could cover the main individual variation within the local population to a certain extent. However, it should be noted that for wild populations with high interindividual differences, three replicates still have limitations in representing the overall population characteristics, which will be further discussed in the limitations section.

Since the freshness of feces directly affects DNA yield and the accuracy of high-throughput sequencing results, during field sampling, active great bustards were first observed. After they left, we promptly arrived at the activity area, selected fresh and moist fecal samples, and placed them into sterilized 50 mL sampling tubes, which were temporarily stored in a −4°C mobile ice box. All samples were transported in mobile ice boxes throughout the field period and, upon being transported back to the laboratory within 12 h, were immediately transferred to a −80°C refrigerator for frozen storage, pending subsequent DNA extraction.

### 2.2. DNA Extraction and PCR Amplification

Microbial DNA was extracted from fecal samples using the E.Z.N.A. fecal DNA Kit (United States) following the manufacturer's protocols. The V3–V4 region of the bacteria 16S ribosomal RNA (*16S rRNA*) gene was amplified by PCR using primers 341F 5⁣′-barcode-CCTAYGGGRBGCASCAG-3⁣′ and 806 R 5⁣′-GGACTACNNGGGTWTCTAAT-3⁣′, where barcode is an eight-base sequence unique to each sample [37].

### 2.3. Library Construction and Sequencing

Purified PCR products were quantified by Qubit3.0 (Life Technologies) and every 24 amplicons whose barcodes were different were mixed equally. The pooled DNA product was used to construct Illumina Pair-End libraries following Illumina's genomic DNA library preparation procedure. Then, the amplicon library was paired-end sequenced (2 × 300) on an NGS platform (Shanghai BIOZERON Biotech. Co. Ltd) according to the standard protocols.

The raw reads were deposited into the NCBI Sequence Read Archive (SRA) database (BioProject Accession: PRJNA1298890 and SRA Accessions: SRR34780477–SRR34780524).

### 2.4. Bioinformatics Analysis

Raw fastq files were first demultiplexed using Trimmomatic v0.39 [38] and in-house perl scripts according to the barcode sequence information for each sample with the following criteria: (i) The 300 bp reads were truncated at any site receiving an average quality score < 20 over a 10 bp sliding window, discarding the truncated reads that were shorter than 50 bp; (ii) exact barcode matching, allowing two nucleotide mismatches in primer matching, and removal of reads containing ambiguous characters were implemented; (iii) only sequences that overlapped longer than 10 bp were assembled according to their overlap sequence. Reads which could not be assembled were discarded.

Passed sequences were dereplicated and subjected to the DADA2 algorithm (QIIME2 v2023.7 recommended) to identify indel mutations and substitutions [39]. The trimming and filtering were performed on paired reads with a maximum of two expected errors per read (maxEE = 2), following the parameter settings optimized for *16S rRNA* gene V3–V4 region sequencing [40]. After merging paired reads and chimera filtering (removing chimeric sequences using the “consensus” method in DADA2), the phylogenetic affiliation of each *16S rRNA* gene sequence (herein called amplicon sequence variants, i.e., ASVs) was analyzed by the UCLUST algorithm (v1.2.22q) [41] against the Silva (SSU138.1) *16S rRNA* database using a confidence threshold of 80% [42], consistent with standard taxonomic assignment protocols for avian gut microbiota studies [43].

### 2.5. Alpha and Beta Diversity Analyses

The rarefaction analysis based on Mothur v.1.48.1 [44] was conducted to reveal the diversity indices, including the Chao1 (estimating species richness), ACE (abundance-based coverage estimator), Simpson (measuring community evenness and diversity), and Shannon (quantifying species diversity) diversity indices with rarefaction curves generated by subsampling to the minimum number of reads across all samples (18,523 reads) to avoid bias from uneven sequencing depth [45]. The beta diversity analysis was performed using the Euclidean distance matrix to Bray–Curtis (quantifying community composition dissimilarity based on relative abundance) or weighted/unweighted UniFrac (incorporating phylogenetic relationships) distance matrices to compare the results of the nonmetric multidimensional scaling (NMDS) with the vegan v2.6-4 community ecology package in R v4.3.1 [46] with NMDS stress values < 0.2 indicating reliable ordination [47].

### 2.6. Difference Analysis

Multivariate analysis of variance (MANOVA) was conducted using the R stats v4.3.1 package to further confirm the observed differences in diversity indices, with post hoc Tukey's HSD test for pairwise comparisons [48]. The R pheatmap v1.0.12 package was applied to visualize the relationships through correlation heatmaps, with row/column clustering based on Euclidean distance and complete linkage [49]. Three complementary nonparametric multivariate statistical tests (Adonis, ANOSIM, and MRPP) [50–52] were performed using the R vegan v2.6-4 package to assess the statistically significant difference of bacterial communities' diversity indices between samples: Adonis (permutational MANOVA, 999 permutations) to test the proportion of variance explained by grouping factors, ANOSIM (analysis of similarities, 999 permutations) to compare within- and between-group dissimilarities, and MRPP (multiresponse permutation procedure, 999 permutations) to evaluate differences in community composition [53]. Differences were considered significant at *p* < 0.05. Venn diagrams were drawn using the online tool “Draw Venn Diagram” (https://bioinformatics.psb.ugent.be/webtools/Venn/) to analyze overlapped and unique OTUs during the treatment processes, with OTUs defined as clusters of sequences with ≥ 97% similarity using UCLUST [41]. One-way permutational analysis of variance (PERMANOVA) was performed using the R vegan v2.6-4 package (999 permutations) to assess the statistically significant effects of treatment processes on bacterial communities, with pairwise PERMANOVA for post hoc comparisons [54].

### 2.7. Functional Prediction of the Microbial Genes

The Phylogenetic Investigation of Communities by Reconstruction of Unobserved States (PICRUSt2 v2.5.1) [55] program based on the Kyoto Encyclopedia of Genes and Genomes (KEGG) database (v107) was used to predict the functional alteration of microbiota in different samples, with the “picrust2_pipeline.py” script and default parameters (including *16S rRNA* gene copy number correction and metagenome prediction) [56]. The ASV data obtained were used to generate BIOM files formatted as input for PICRUSt2 with the make.biom script usable in the Mothur v.1.48.1 [44]. Tax4Fun2 v1.1.7 [57] (R package) is used for predicting functional profiles and functional gene redundancies of prokaryotic communities from *16S rRNA* sequences using the SILVA SSU138 database and BLAST+ v2.14.0 for sequence alignment (e-value cutoff of 1e − 5) [57, 58]. Predictions of the metabolic capabilities of microbiota were performed with functional annotation of prokaryotic taxa (FAPROTAX v1.2.4) [59] using *16S rRNA* sequencing data, with the default database and parameter settings (minimum read count threshold of 10 for taxon inclusion) [60].

## 3. Results

### 3.1. Intestinal Microbial Composition of Great Bustards

This study systematically characterized the compositional characteristics of intestinal microbiota in great bustards *O. tarda* across six taxonomic levels: phylum, class, order, family, genus, and species (Supporting Information3: Table S2). Results revealed that their intestinal microbiota structure exhibits high diversity and marked heterogeneity.

Regarding overall taxonomic coverage, a total of 34 phyla, 70 classes, 169 orders, 281 families, 624 genera, and 226 species were detected in the feces of bustard collected from SX Province, HB Province, and the NMG in China (Supporting Information 3: Table S2), indicating that the intestinal microbiota of *O. tarda* comprises abundant taxonomic units and generally exhibits high species diversity. At each taxonomic level, significant differences were observed in the number of taxonomic units among samples. For instance, 28 phyla were detected in sample DL3_2, whereas only nine were identified in samples such as HZ1_1 and HZ2 (including HZ2_1, HZ2_2, and HZ2_3), with the former being 3.11 times that of the latter (*p* < 0.05). At the genus level, sample DL3_2 contained 385 genera—significantly more than the 126 genera in sample HZ1_1 (3.05-fold higher) (*p* < 0.01). At the species level, sample DL3_2 harbored 78 species, while sample NMG_2 contained only 12, representing a 6.5-fold difference (*p* < 0.001). Across all samples, significant variations in intestinal microbial richness were observed at all taxonomic levels (Supporting Information 3: Table S2).

The number of taxonomic units in each sample generally increased from higher to lower taxonomic levels, consistent with the hierarchical logic of microbial taxonomy (i.e., more specific taxonomic units correspond to a greater number of identifiable entities). For example, sample HZ1_2 contained 14 phyla, 26 classes, 60 orders, 87 families, and 149 genera, while sample DL2_3 contained 16 phyla, 24 classes, 65 orders, 98 families, and 171 genera (Supporting Information 3: Table S2). Notably, the number of species-level taxonomic units was generally small (10–78), with the number of species in each sample being less than half that of genera.

**Figure 2:**
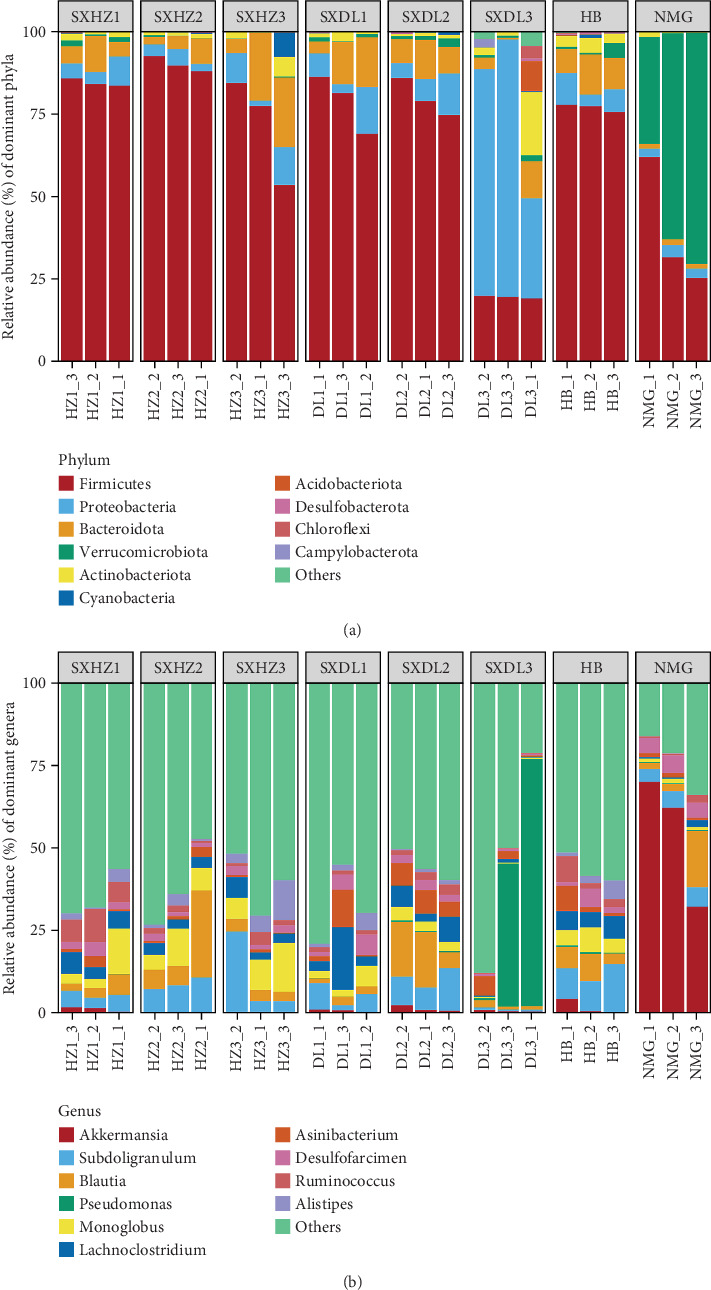
Microbiota structure in the feces of great bustards *Otis tarda*. (a) Phylum-level relative abundances. (b) Genus-level relative abundances.

At the phylum level, Firmicutes, Proteobacteria, and Bacteroidota were the core dominant phyla across most samples (Figure 2a), with their cumulative relative abundance frequently approaching or exceeding 75%. Firmicutes typically accounted for the highest proportion; for example, in the SXHZ, SXDL (except SXDL3), and HB groups, Firmicutes constituted over half of the microbiota (*p* < 0.05). While Proteobacteria and Bacteroidota exhibited variable but notable abundances, Verrucomicrobiota, Actinobacteriota, Desulfobacterota, and Cyanobacteria contributed secondarily. Acidobacteriota, Chloroflexi, Campylobacterota, and the “Others” category maintained relatively low proportions (< 25%). Notably, SXDL3 exhibited a distinct profile, characterized by an unusually high abundance of Proteobacteria—differing from the SXHZ, SXDL (except SXDL3), and HB groups—indicating a unique microbial niche within this group (*p* < 0.01). Samples from NMG showed a striking shift: While Firmicutes remained dominant, abundances of Proteobacteria and Bacteroidota decreased, accompanied by an increased proportion of Verrucomicrobiota, suggesting unique environmental drivers shaping phylum-level composition in this population (*p* < 0.001).

At the genus level, *Lachnoclostridium*, *Subdoligranulum*, *Blautia*, *Monoglobus*, *Ruminococcus*, *Alistipes*, *Desulfofarcimen*, *Pseudomonas*, and *Asinibacterium* typically exhibited relatively high proportions in many samples (Figure 2b). Certain samples displayed distinct profiles with exceptionally high abundances of specific genera, signifying unique microbial niches. For example, all NMG samples exhibited an unusually high abundance of *Akkermansia* (*p* < 0.05), while *Pseudomonas* was prominently abundant in DL3_2 and DL3_3 (*p* < 0.01).

In summary, the fecal microbiota of great bustards exhibits substantial diversity across taxonomic levels, with significant variations among groups/samples. Furthermore, distinct core taxa dominate at both the phylum and genus levels, and their distribution patterns may likely be associated with factors such as individual differences and the living environments of great bustards (inferred from sampling site habitat characteristics, as direct environmental parameter measurement was not conducted).

### 3.2. Characteristic Analysis of Shared Microbial Taxa

**Figure 3:**
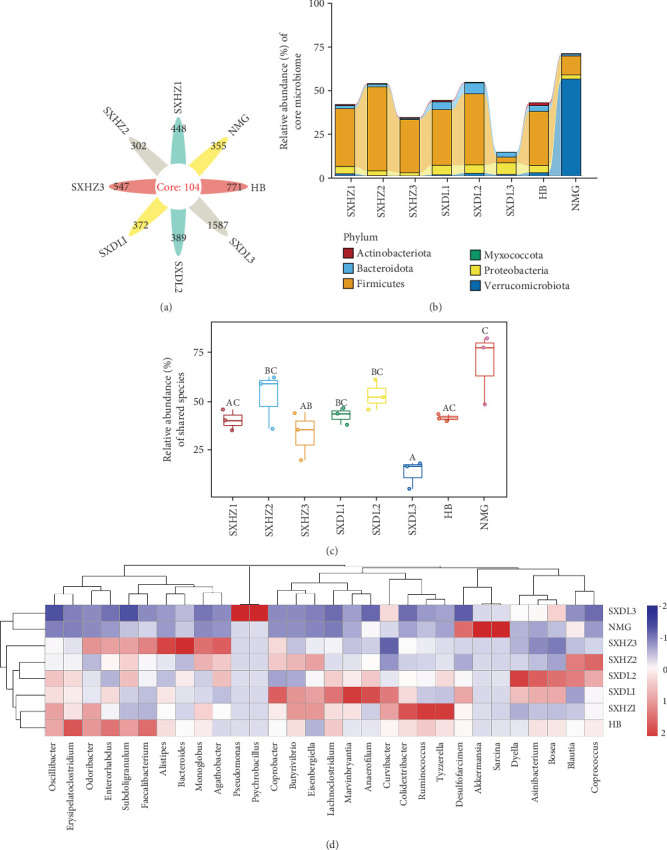
Multidimensional analysis of the fecal microbiota characteristics of great bustards *Otis tarda*. (a) Distribution of core shared taxa and unique taxa among different groups. (b) Relative abundances of core shared taxa at the phylum level among different groups. (c) Differences in relative abundances of core shared taxa at the phylum level among different groups. (d) Heatmap of abundances of core shared taxa at the genus level for core taxa among different groups.

In terms of the distribution of shared taxa among groups/samples, the number of core shared taxa across all groups was 104 (across all samples was 41) (Figure 3a, Supporting Information 1: Figure S1A, and Supporting Information 3: Tables S3 and S4). ASV data (Supporting Information 3: Tables S3 and S4) showed that the core shared taxa were mainly affiliated with the previously identified dominant phyla and genera. Sixty-eight of 104 ASVs belonged to the phylum Firmicutes, among which the majority (63 ASVs) were classified into the class Clostridia, with the remaining distributed in Bacilli (four ASVs) and Desulfotomaculia (one ASV). Within Clostridia, the dominant orders included Lachnospirales (e.g., family Lachnospiraceae) and Oscillospirales (e.g., family Ruminococcaceae and Oscillospiraceae). Twenty of 104 ASVs were classified into the phylum Proteobacteria, predominantly belonging to Alphaproteobacteria (11 ASVs) and Gammaproteobacteria (nine ASVs), with Rhizobiales and Burkholderiales as the main orders. Eight of 104 ASVs belonged to Bacteroidota, mainly within Bacteroidia (seven ASVs) and Chitinophagales (one ASV); five were in the phylum Actinobacteriota, primarily affiliated with Coriobacteriia (four ASVs); and the other three ASVs were in the phyla Verrucomicrobiota, Myxococcota, and Desulfobacterota, respectively.

Among different groups (i.e., SXHZ1–SXHZ3, SXDL1–SXDL3, HB, and NMG), the relative abundance of shared species ranged from 25% to 75%, with statistically significant differences between groups (Figure 3b). At the phylum level, Firmicutes showed the highest relative abundance in SXHZ2 (47.55%) and the lowest in NMG (10.67%) (*p* < 0.01), while Verrucomicrobiota exhibited an opposite pattern, with the highest abundance in NMG (54.83%) and negligible levels in other groups (e.g., 0.05% in SXHZ3) (*p* < 0.001). Proteobacteria and Bacteroidota were relatively abundant in SXDL samples, with Proteobacteria reaching 6.63% in SXDL3 and Bacteroidota peaking at 6.16% in SXDL2 (Figure 3b) (*p* < 0.05).

Further analysis of intragroup variations (Supporting Information 3: Table S5) revealed fluctuations in phylum relative abundance among replicate samples within groups. For example, in HZ1, Firmicutes ranged from 32.28% (HZ1_1) to 33.86% (HZ1_3), and Verrucomicrobiota varied more notably (0.45% in HZ1_2 vs. 1.74% in HZ1_3) (*p* < 0.1). SXHZ2 showed marked Firmicutes differences (32.55% in HZ2_1 vs. 58.21% in HZ2_2) and Proteobacteria variations (1.84%–3.63%) (*p* < 0.05). In SXDL1, Bacteroidota (0.49%–11.38%) and Proteobacteria (1.68%–9.75%) fluctuated significantly (*p* < 0.05). SXDL3 exhibited extreme variability in Proteobacteria (1.19% vs. 12.05%) and Bacteroidota (0.36% vs. 5.71%), consistent with its low Firmicutes (3.04%–3.42%) (*p* < 0.001). HB showed large Verrucomicrobiota (0.49%–4.50%) and Bacteroidota (0.83%–7.49%) variations (*p* < 0.05). NMG, dominated by Verrucomicrobiota, displayed intragroup differences in this phylum (32.33% vs. 69.93%), while Firmicutes remained stable (8.52%–13.12%) (*p* < 0.05).

Multiple comparison analysis of intergroup differences (Figure 3c) further confirmed significant variations: The relative abundance of shared species between NMG and SXDL3 showed an extremely significant difference (*p* < 0.001), and significant differences were observed between SXDL3 and SXHZ2 (*p* = 0.00509) as well as between NMG and SXHZ3 (*p* = 0.00964). Marginally significant differences (*p* < 0.1) were found between SXDL3 and SXHZ1 (*p* = 0.07895), NMG and SXHZ1 (*p* = 0.05159), HB and SXDL3 (*p* = 0.06252), NMG and SXDL1 (*p* = 0.08621), and NMG and HB (*p* = 0.06511).

### 3.3. Diversity Characteristics of the Intestinal Microbiota

An *α*-diversity analysis (Supporting Information 3: Table S6) covering indices such as species richness, diversity, and evenness was conducted on the intestinal microbiota of great bustards. Results showed that in terms of species richness, the SXDL3 group contained the highest number of microbial species (748.67 ± 787.90), but with an extremely large standard deviation (SD) within the group (*p* < 0.05). The HB group (655.00 ± 130.10) ranked second, with good stability within the group, while the NMG group (305.33 ± 24.54) had the lowest species richness and high consistency within the group (*p* < 0.05). The SXHZ series groups (SXHZ1–3) exhibited moderate species richness (479.00 ± 148.62 in SXHZ1, 358.33 ± 10.02 in SXHZ2, and 469.33 ± 120.57 in SXHZ3), with certain differences within the group (*p* < 0.1).

The HB group had the highest Shannon index (4.96 ± 0.10) and Simpson index (0.98 ± 0.00), indicating the highest microbial diversity (Supporting Information 3: Table S6) (*p* < 0.05). The NMG group had the lowest Shannon index (2.52 ± 0.75) and Simpson index (0.66 ± 0.19) (*p* < 0.05). The NMG group had the lowest Shannon index (2.52 ± 0.75) and Simpson index (0.66 ± 0.19), which were significantly lower than those of other groups (*p* < 0.05, Supporting Information 3: Table S6). The Shannon indices of microbiota in feces of great bustards from Huazhou District, SX Province (SXHZ1–3), ranged from 4.18 to 4.56, and the Simpson indices were mostly 0.95-0.97, with differences within the group and from other groups, reflecting the characteristics of intestinal microbial diversity of great bustards in different periods in Huazhou District (*p* < 0.05). For microbiota in feces of great bustards from Dali County, SX Province (SXDL1–3), the Shannon indices ranged from 3.76 to 4.44 and the Simpson indices from 0.81 to 0.97 (*p* < 0.05). The SXDL3 group showed obvious fluctuations in indices due to large individual differences, reflecting variations in intestinal microbial diversity of great bustards in different periods in Dali County.

In terms of evenness, the microbiota in feces of great bustards from HB Province had the highest Pielou *J* index (0.77 ± 0.02), with the most uniform species distribution; the microbiota in feces of great bustards from NMG had the lowest Pielou *J* index (0.44 ± 0.13) (*p* < 0.001). The Pielou *J* indices of microbiota in feces of great bustards from Huazhou District, SX Province, ranged from 0.71 to 0.74 and those of the SXDL series groups from 0.58 to 0.74 (*p* < 0.05). Differences between groups may be associated with factors such as habitat environment (inferred from sampling site vegetation) and sampling time, potentially reflecting the characteristics of community species distribution in samples from different regions and periods.

Overall, the *α*-diversity indices of microbiota in feces of great bustards from NMG were the lowest, with high stability within the group; the microbial diversity characteristics in feces of great bustards from HB Province were the best, with good consistency within the group; the SXDL3 group had a high average value but significant differences within the group; except for SXDL3, the *α*-diversity of other groups of microbiota in feces of great bustards from Huazhou District and Dali County, SX Province, was at a moderate level. These differences may be related to factors such as the habitat environment, individual status, and sampling time.

This study further explored community differentiation patterns from multiple dimensions using weighted UniFrac, unweighted UniFrac, and Bray–Curtis distance matrices (Supporting Information 3: Table S7) to quantify the similarity of community structures between samples (the smaller the distance, the more similar the community structure). Results showed that after grouping samples into SXHZ, SXDL, HB, and NMG, intragroup similarity was significantly higher than intergroup similarity, with distinct differences in microbiota between different populations. Among them, the NMG group stood out in the weighted UniFrac distance; the intragroup sample distance was extremely small (e.g., only 0.01 between NMG_1 and NMG_2); the Bray–Curtis distance was also significantly low (0.10 between NMG_1 and NMG_2) (*p* < 0.001).

Intragroup distances in the SXHZ series (SXHZ1–3) and SXDL series (SXDL1–3) were smaller than intergroup distances (e.g., the weighted distance between HZ1_1 and HZ1_2 was 0.04) (*p* < 0.001). Additionally, differences in intragroup distances were observed between samples from different time points in Huazhou District and Dali County (*p* < 0.05). The intragroup similarity of the HB group, although slightly lower than that of NMG, remained higher than the intergroup similarity (*p* < 0.001). The special sample DL3_2 showed significantly higher distances from other samples in all three matrices, with unweighted and Bray–Curtis distances often exceeding 0.9 (*p* < 0.001).

In summary, the intestinal microbiota structure of great bustards exhibits population specificity. The NMG group has the strongest stability, and the DL3_2 sample has unique community characteristics. Meanwhile, due to differences in sampling time and regions, the SXHZ and SXDL series groups show corresponding patterns in intragroup community structure consistency and differences from other groups.

**Figure 4:**
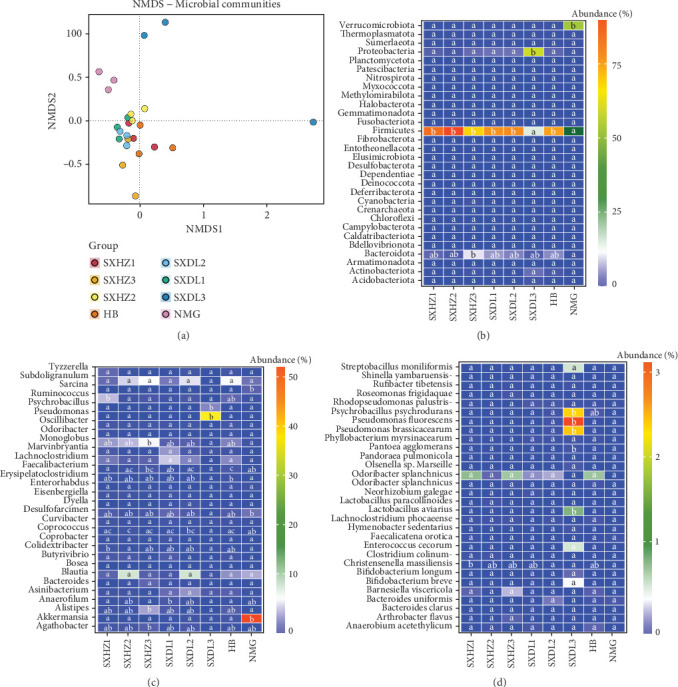
Characterization of the fecal microbiota of great bustards *Otis tarda*. (a) Similarity of great bustard intestinal microbiota structures. (b) Heatmaps of relative abundance differences at phylum levels. (c) Heatmaps of relative abundance differences at genus levels. (d) Heatmaps of relative abundance differences at species levels.

This study visualized the similarity of great bustard intestinal microbiota structures using NMDS analysis (Figure 4a). In terms of intragroup aggregation, groups such as SXHZ1–3, SXDL1–2, and HB were relatively clustered, indicating high similarity and strong stability in microbiota structures among intragroup samples (*p* < 0.05). In contrast, the SXDL3 and NMG groups were scattered (e.g., SXDL3 contained isolated points far from the center), showing significant individual differences in community structure (*p* < 0.05).

In terms of intergroup differentiation, the NMG group was overall far from other groups, indicating significant differences in its microbiota from other populations (*p* < 0.001); the SXDL3 group was scattered with some points far from the center, reflecting both intragroup differences and obvious differentiation from other groups (*p* < 0.05); the HB group was relatively clustered with overlaps with most groups, indicating certain similarities in community structure with other groups while maintaining uniqueness. These results intuitively verify the “intragroup similarity, intergroup difference” pattern of great bustard intestinal microbiota; reflect the influence of factors such as habitat, population niche, sampling region, and time on community structure; and provide visual evidence for analyzing microbe–host–environment interactions.

The heatmap of relative abundance and significance of differences in intestinal microbial phyla of great bustards (Figure 4b) revealed significant differences in dominant phyla. Firmicutes had high abundance (50%–75%) in SXHZ1–3, SXDL1–2, and HB groups, showing significant differences from SXDL3 and NMG, and were the core driver of community structure (*p* < 0.05). Verrucomicrobiota showed significant differences between the NMG group and other groups, being a uniquely high-abundance phylum in NMG and reflecting population specificity (*p* < 0.001). Proteobacteria showed significant differences between the SXDL3 group and other groups, reflecting community differences in special individuals (e.g., DL3_2) in SXDL3 (*p* < 0.01). Additionally, fluctuations in the abundance of dominant phyla among intragroup samples from different time points in Huazhou District and Dali County reflect the impact of regional and temporal factors on phylum distribution. Low-abundance phyla (e.g., Thermoplasmatota) had low abundance with no intergroup differences, belonging to “background” microorganisms.

Combined with multiomics data, the NMG group exhibits uniqueness due to Verrucomicrobiota and other phyla; the SXDL3 group shows individual differences due to the abnormal enrichment of Proteobacteria; the HB group shows significant differences in Firmicutes abundance from some groups. These results verify the pattern of microbiota differences between different populations, identify core differential phyla for analyzing the “microbe–host adaptation mechanism,” and help understand the ecological association between intestinal flora and hosts, as well as the shaping of phylum-level flora structure by regional and temporal factors.

The heatmap of relative abundance and significance of differences in intestinal microbial genera of great bustards (Figure 4c) revealed significant differences in the abundance of dominant genera. For example, *Akkermansia* showed significant differences between the NMG group and other groups, being a uniquely high-abundance genus in NMG and reflecting population specificity (*p* < 0.001); *Pseudomonas* showed significant differences between the SXDL3 group and other groups, reflecting community differentiation in special individuals of SXDL3 (*p* < 0.01). Fluctuations in the abundance of dominant genera among intragroup samples from different time points in Huazhou District (SXHZ1–3) and Dali County (SXDL1–3) further reflect the impact of regional and temporal factors on genus-level community structure.

*Faecalibacterium* had relatively high abundance in the SXHZ series (SXHZ1–3) with intergroup differences, suggesting it is a dominant genus with fluctuating abundance in the SXHZ population and reflecting its distribution characteristics in samples from different time points in Huazhou District (*p* < 0.05); *Coprococcus* showed significant intra- and intergroup abundance differences in the SXHZ and SXDL series, which may be related to population niche, sampling region, and time (*p* < 0.05). Low-abundance genera (e.g., *Bosea* and *Eisenbergiella*) are widespread as “background” microorganisms with little contribution to community differentiation.

The heatmap of relative abundance and significance of differences in intestinal microbial species of great bustards (Figure 4d) revealed differential species in a low-abundance background. Most species had abundances of nearly 0%–1% in all groups, reflecting the overall low-abundance characteristic of microbiota at the species level. However, some species showed intergroup abundance differences; for example, *Pseudomonas fluorescens* had high abundance (approximately 3%) in the SXDL3 group with significant differences from other groups, being a uniquely high-abundance species in SXDL3 and reflecting community differentiation in special individuals of this group (*p* < 0.01); *Lactobacillus aviarius* had an abundance of approximately 1.5% in the HB group with significant differences from other groups, reflecting the unique community characteristics of HB (*p* < 0.05). Fluctuations in species-level abundance among intragroup samples from different time points in Huazhou District and Dali County reflect the impact of regional and temporal factors on species distribution.

In terms of population-specific species distribution, most species in the NMG group had low abundance, with only a few showing no significant differences from other groups, indicating a relatively uniform and low-abundance species-level community (*p* < 0.05); some species in the SXHZ and SXDL series (e.g., *Psychrobacillus psychrodurans* in SXDL3) showed intra- and intergroup abundance fluctuations, reflecting the shaping of species-level communities by intrapopulation niche differences, region, and time (*p* < 0.05).

### 3.4. Functional Prediction of Microbiota

This study utilized three microbiota function prediction tools, namely, PICRUSt2, Tax4Fun, and FAPROTAX, to analyze the functions of the intestinal microbiota of great bustards sampled from different regions (HB, SXDL, SXHZ, and NMG). The characteristics of the community functions were revealed from the aspects of the distribution of major functional categories, subfunctional categories, and ecological association. In terms of the major functional categories (Supporting Information 2: Figure S2A,B), “metabolism” is the core functional module. All groups showed a high abundance with no differences among groups. The overall abundance of the “human diseases function” is relatively low, and only the samples in the SXDL3 group showed significant differences from other groups (*p* < 0.05). The functions of “Genetic Information Processing and Environmental Information Processing” showed significant differences among groups, with the former showing obvious differences in the SXDL3 group compared to other groups (*p* < 0.05).

At the level of subfunctional categories and ecological associations (Supporting Information 2: Figure S2C), “chemoheterotrophy” as a dominant ecological strategy showed a high abundance in all groups with no differences among components. For the “fermentation”, the SXDL3 group showed obvious differences compared to other groups with a decreased abundance (*p* < 0.05). Among the special functions, the “animal parasites or symbionts” in the NMG group showed obvious differences compared to other groups (*p* < 0.05). The “aerobic chemoheterotrophy” showed obvious differences and a slightly higher abundance only in the NMG and SXDL3 groups compared to other groups (*p* < 0.05), with slightly higher abundance in the NMG and SXDL3 groups compared to other groups.

In summary, the functions of the intestinal microbiota of great bustards have both conservation and specificity: Basic functions, such as metabolism, maintain the essential needs for the host's survival. The functions of “Genetic Information Processing and Environmental Information Processing” respond to changes in the regional–temporal environment. “Chemoheterotrophy” is the core ecological strategy, and the differentiation of functions such as “fermentation” is associated with the host–environment interaction. This study analyzes the putative “microbe–host–environment” interaction mechanism at the functional level. In the future, by combining host physiology and environmental factors, we can further explore the impact of predicted functional differences on the ecological adaptation of great bustards, providing theoretical support for microbial ecology in the protection of endangered birds.

## 4. Discussion

The intestinal microbiota of animals is a dynamic ecosystem shaped by intricate interactions between host physiology, ecological traits, and environmental factors [1, 61]. For bustards, prior research has established foundational knowledge of gut microbial composition and documented diverse aerobic bacteria and fungi in three captive bustard species [12], while Lu et al. [13–15] clarified core microbiota, captivity impacts, and overwintering dynamics of the great bustard (*O. tarda dybowskii*). This study, using fecal eDNA metabarcoding, revealed significant geographical differences (across HB, NMG, and SX) and temporal dynamics (monthly scales in SX) in the intestinal microbiota of the endangered great bustard *O. tarda* (Figure 1), filling critical gaps in understanding microbial adaptation to diverse habitats. Below, we integrate our findings with the species' ecological traits and existing literature to interpret the drivers, functional significance, and conservation implications of these microbial patterns.

### 4.1. Drivers of Microbial Diversity Differences: Geography, Climate, and Individual Variation

The *β*-diversity analysis confirmed distinct microbiota structures among populations from HB, NMG, and SX, with intragroup similarity significantly higher than intergroup similarity (Figure 4a). This geographical partitioning aligns with the hypothesis that habitat-specific environmental factors—particularly vegetation type and food resources (inferred from habitat surveys)—are primary drivers [19, 62, 63]. A recent meta-analysis of avian gut microbiota further supported this, showing that 62% of geographical variation in passerine gut communities could be explained by differences in local plant diversity and insect availability [20]. This pattern is not limited to small birds: in large terrestrial avians like the African ostrich (*Struthio camelus*), gut microbiota diverged between savanna and desert populations, with desert individuals enriched in taxa (e.g., *Desulfovibrio*) that enhance water retention—similar to how NMG's grassland great bustards are enriched in Verrucomicrobiota for stress tolerance.

The unique enrichment of *Akkermansia* in NMG and *Pseudomonas* in SXDL3 samples may be driven by habitat-specific environmental or biological factors (e.g., diet and climate). The NMG population, inhabiting grassland ecosystems, exhibited a unique microbial profile dominated by Verrucomicrobiota (notably *Akkermansia*), in contrast to the Firmicutes-rich communities in SX (farmland habitats) and HB (Figures 2a and 3b). The lowest Pielou *J* index (0.44 ± 0.13) in NMG suggests that a few species (e.g., *Akkermansia*) may dominate the microbiota, while most species have extremely low abundance. This divergence aligns with prior findings on bustard microbiota: Wintering great bustards relying on farmland diets (rich in starch) maintain high Firmicutes abundance, while our study extends this by showing that grassland habitats (low-nutrient and high-fiber diets) select for Verrucomicrobiota [14]. This divergence likely stems from dietary differences—great bustards in NMG primarily feed on grassland herbs and seeds [36], while those in SX rely more on crops (e.g., wheat and oilseed rape) and associated insects [32]. This divergence may stem from dietary differences—great bustards in NMG are reported to primarily feed on grassland herbs and seeds [36], while those in SX are reported to rely more on crops (e.g., wheat and oilseed rape) and associated insects [32] (diet data from literature, not direct measurement in this study)—though both food types are common to many Gruiformes rather than being specialized to this species. Grassland diets, typically higher in recalcitrant fibers and lower in nutrient density [64, 65], may select for microbial taxa like *Akkermansia* (within Verrucomicrobiota) that are associated with potential enhancement of intestinal barrier integrity and putative efficient energy utilization under resource limitation [66], a pattern consistent with the research observation that environmental stress (e.g., captivity) shifts great bustard microbial functional traits [15]. Conversely, farmland diets (rich in starch and cultivated plants) favor Firmicutes and Bacteroidota—taxa well-documented in great bustards [13, 14] for their ability to degrade complex carbohydrates [20, 21].

The microbiota of SXHZ and SXDL showed measurable shifts across December (SXHZ1 and SXDL1), January (SXHZ2 and SXDL2), and March/February (SXHZ3 and SXDL3) (Figure 3b). This aligns with the finding that great bustard gut microbiota changes during late winter (March) due to dietary deterioration, though our study further resolves monthly-scale dynamics [14]. For other avian species, such short-term temporal shifts are also linked to fitness: In the blue tit (*Cyanistes caeruleus*), weekly changes in gut microbiota during winter correlated with increased expression of host immune genes, suggesting microbial dynamics support seasonal physiological adjustments [21]. In waterfowl like the mallard (*A. platyrhynchos*), monthly microbiota shifts tracked changes in aquatic plant phenology, with increased abundance of fiber-degrading taxa (e.g., Ruminococcaceae) when tough emergent plants dominated [22]. These temporal fluctuations correspond to changes in food availability (inferred from seasonal vegetation dynamics [29]): For example, crop residues are reported to gradually diminish, while winter weeds and overwintering insects become more prominent by March [29]. Such dietary shifts likely drive changes in microbial functional groups—for example, increased abundance of fiber-degrading taxa (e.g., Lachnospiraceae) in later months as reliance on tough plant material rises. The relative stability of *α*-diversity in SXHZ compared to SXDL suggests that Huazhou's more consistent agricultural landscapes may buffer microbial fluctuations, whereas Dali's riparian habitats (with variable food resources) induce greater variability—complementing the observation that stable habitats reduce overwintering microbial variability [14]. The significant intergroup differences (e.g., NMG vs. SXDL3, *p* < 0.001) and intragroup variations in shared taxa abundance suggest that microbiota divergence is shaped by both group-specific factors (geography, ecology, and time) and individual/population differences, reflecting the complexity of microbiota dynamics.

### 4.2. Ecological Functions of Core Taxa: Adaptations to Habitat-Specific Challenges

The dominant microbial taxa identified—Firmicutes, Bacteroidota, Verrucomicrobiota, and Proteobacteria (Figures 2a and 3b)—exhibit functional traits that align with the great bustard's ecological needs, providing mechanistic links between microbiota and host adaptation.

As the most abundant phyla in SX and HB populations (Firmicutes and Bacteroidota), these taxa are consistent with prior findings on great bustards: identifying them as core phyla in wild wintering populations, noting their role in degrading plant fibers and metabolizing complex carbohydrates—functions critical for utilizing farmland diets (e.g., wheat starch) [13, 14]. Their high abundance in farmland habitats (SX) likely reflects the great bustard's reliance on crops (e.g., wheat seedlings), which require efficient breakdown of cellulose and starch to support energy storage during winter. The ratio of Firmicutes to Bacteroidota, a putative marker of potential energy harvest efficiency [6], was consistently high in SXHZ and SXDL—mirroring the observation of a high Firmicutes/Bacteroidota ratio in wintering great bustards—and suggesting [13] a putative adaptive strategy associated with potential maximization of energy acquisition in resource-rich but temporally variable agricultural landscapes.

The extreme enrichment of Verrucomicrobiota (up to 54.83% in NMG) is a striking feature of the grassland population [67, 68] and a novel finding compared to prior great bustard studies [13–15], which focused on farmland or captive populations. *Akkermansia*, a key genus within this phylum, has been associated with potential enhancement of intestinal barrier integrity and putative reduction of energy expenditure by regulating mucus degradation [7, 9]. In NMG's arid, cold grasslands, where food is scarce and thermoregulatory costs are high, this trait would be advantageous—complementing the finding that microbial taxa linked to stress tolerance are enriched in challenging environments [15]. A stronger gut barrier minimizes energy loss from inflammation, while efficient nutrient absorption offsets limited food intake [69–71]. This aligns with reports of *Akkermansia* enrichment in animals adapting to harsh environments [72], suggesting it is a signature of the great bustard's adaptation to grassland stressors.

The spike in Proteobacteria in SXDL3 (e.g., *Pseudomonas*) may reflect local environmental conditions, such as increased humidity in riparian habitats or exposure to environmental pathogens—similar to the results that *Pseudomonas* is associated with clinical cases (e.g., conjunctivitis and abscesses) in captive bustards, though our study finds it in wild individuals as a response to environmental fluctuations. Proteobacteria are metabolically diverse and chemotactic, enabling rapid responses to fluctuating resources or stressors [8, 31]. Their enrichment could represent a putative “resilience strategy” potentially allowing great bustards to cope with transient environmental changes (e.g., sudden rainfall altering food availability or pathogen loads) during late winter, extending the finding that late-winter microbial shifts are linked to putative environmental stress [14].

The high abundance of “chemoheterotrophy” across all groups suggests it is a core ecological strategy for energy provision via organic compound utilization. The decreased “fermentation” function in SXDL3 may indicate reduced short-chain fatty acid–producing flora, potentially affecting intestinal health and energy utilization. The unique “animal parasites or symbionts” function in NMG may be linked to local pathogen/symbiont distribution, while elevated “aerobic chemoheterotrophy” in NMG and SXDL3 may reflect adaptation to habitat-specific oxygen partial pressure.

### 4.3. Conservation Implications: Microbiota as a Tool for Adaptive Management

Low microbial diversity in NMG (e.g., the lowest Shannon index) may indicate food resource uniformity in grasslands—consistent with the observation that homogeneous environments reduce great bustard microbial diversity [15]—suggesting the need to restore plant diversity to potentially support microbial and host resilience. Conversely, high diversity in HB (stable and mixed habitats) could serve as a benchmark for habitat quality, aligning with the finding that consistent wintering habitats maintain high microbial diversity in great bustards [13]. In SX's agricultural areas, where Firmicutes (key taxa for degrading complex carbohydrates [13, 14]) dominate, supplementing with fiber-rich plants (e.g., wheat straw and alfalfa hay) during late winter (March)—when crop residues are scarce—could stabilize microbiota (by maintaining Firmicutes abundance) and support energy storage for migration, directly addressing the late-winter dietary deterioration noted in prior studies [14]. In NMG, promoting the growth of *Akkermansia*-friendly plants, drought-tolerant herbs (e.g., *A. frigida* and *S. grandis*) and supplementing with their seeds during food-scarce cold spells may reinforce the gut barrier of great bustards, enhancing their tolerance to harsh conditions—aligning with the role of microbial traits in stress adaptation [15]. Elevated Proteobacteria in SXDL3 (linked to potential pathogens [8]) warrants further investigation into local environmental stressors (e.g., water contamination)—echoing the caution that Proteobacteria like *Pseudomonas* can be associated with bustard diseases—and emphasizing the role of microbiota as early warning indicators of population health.

### 4.4. Limitations and Future Directions

*16S rRNA* sequencing limits species-level identification, particularly for understudied taxa (e.g., unclassified *Clostridia*). Metagenomic sequencing would clarify functional genes, while metabolomics could link microbial activity to host physiology (e.g., short-chain fatty acid production). Species-level identification was limited (10–78 species per sample, < 50% of genus-level units) likely due to challenges in microbial species classification and incomplete database information for avian gut microbiota. Fecal eDNA metabarcoding of plant/insect residues is needed to directly link food resources to microbial shifts, strengthening the causal relationship between diet and microbiota structure. Extending sampling to breeding seasons and migration routes would reveal whether microbial patterns are transient (winter-specific) or consistent across life stages. In vitro culturing of core taxa (e.g., *Akkermansia* from NMG) and host–microbe interaction experiments could confirm their roles in stress tolerance and nutrient metabolism.

Another limitation of this study is the sample size. Each sampling site only included three biological replicates, which may be insufficient to fully capture the interindividual variation of wild great bustard populations. Although the presurvey confirmed the low mobility of the population during the sampling period and the three replicates could reflect the basic characteristics of the local microbiota, wild populations are often affected by factors such as individual age, health status, and foraging range, leading to high interindividual differences. A power analysis was not conducted before the experiment to determine the optimal sample size, which may reduce the statistical power of the results, especially in the analysis of small-scale temporal and spatial differences. In future studies, on the premise of not disturbing the endangered population, we will increase the sample size as much as possible, conduct a preexperiment power analysis to determine the appropriate number of replicates, and combine marking and tracking methods to collect samples from the same individual at different time points to more accurately analyze the dynamic changes of intestinal microbiota.

## 5. Conclusion

In conclusion, this study reveals that the great bustard's fecal microbiota is dominated by Firmicutes, Bacteroidota, and Proteobacteria as core taxa, with Verrucomicrobiota (notably *Akkermansia*) uniquely enriched in NMG. Geographically, significant divergence exists between NMG (grassland) and SX/HB (farmland) populations, which may be driven by habitat-specific diets—grassland herbs (high fiber, inferred from [36]) versus crops (starch-rich, inferred from [32]). Temporally, winter samples (December–March) from SX show dynamic shifts, potentially reflecting fluctuations in food availability (e.g., crop residues and insect abundance, inferred from seasonal habitat surveys [29]). These microbial patterns underscore the gut microbiota's critical role in adaptation. Firmicutes and Bacteroidota facilitate plant fiber digestion in farmlands, while Verrucomicrobiota enhances stress tolerance in NMG's harsh conditions. Such plasticity enables great bustards to cope with habitat and climatic changes. For conservation, the findings highlight microbiota as a bioindicator of habitat quality. Managing grassland plant diversity in NMG and maintaining crop-food stability in SX can support microbial balance. Supplementary feeding targeting fiber-degrading taxa during food-scarce periods may boost resilience, aiding the survival of this endangered species.

## Data Availability

The data that support the findings of this study are available from the corresponding author upon reasonable request. The raw reads of nucleotide sequence were deposited into the NCBI Sequence Read Archive (SRA) database (BioProject Accession: PRJNA1298890 and SRA Accessions: SRR34780477–SRR34780524).

## Supporting Information

Supporting Information 1 Figure S1: Multidimensional analysis of the fecal microbiota characteristics of great bustards *Otis tarda*. (A) Distribution of core shared taxa and unique taxa among different samples. (B) Differences in relative abundances at the phylum level among different samples. (C) Heatmap of abundances at the genus level for core taxa among different samples.

Supporting Information 2 Figure S2: Functional prediction analysis of the great bustard intestinal microbiota based on multiple tools. (A) Distribution of functional categories by PICRUSt2. (B) Distribution of functional categories by Tax4Fun2. (C) Subfunctional categories and ecological associations by FAPROTAX.

Supporting Information 3 Table S1: Sampling information. Table S2: Classification of the intestinal microbiota in the great bustard *Otis tarda*. Table S3: Microorganisms shared among different groups of the great bustard *Otis tarda*. Table S4: Microorganisms shared among different samples of the great bustard *Otis tarda*. Table S5: Relative abundance (percentage) of microorganisms shared among different samples of the great bustard *Otis tarda*. Table S6: Statistical analysis of the *α*-diversity index of intestinal microbiota in different groups of great bustards *Otis tarda*. Table S7: Statistical analysis of the *β*-diversity index of intestinal microbiota in different groups of great bustards *Otis tarda*.
