# Supplementary figures and images for: Geographical Differences and Temporal Dynamics of Intestinal Microbiota in Endangered Great Bustard Otis tarda Based on Environmental DNA Metabarcoding
Source: Int J Microbiol. 2025 Dec 3;2025:5587641. doi: 10.1155/ijm/5587641 (PMC12695410; doi:10.1155/ijm/5587641)

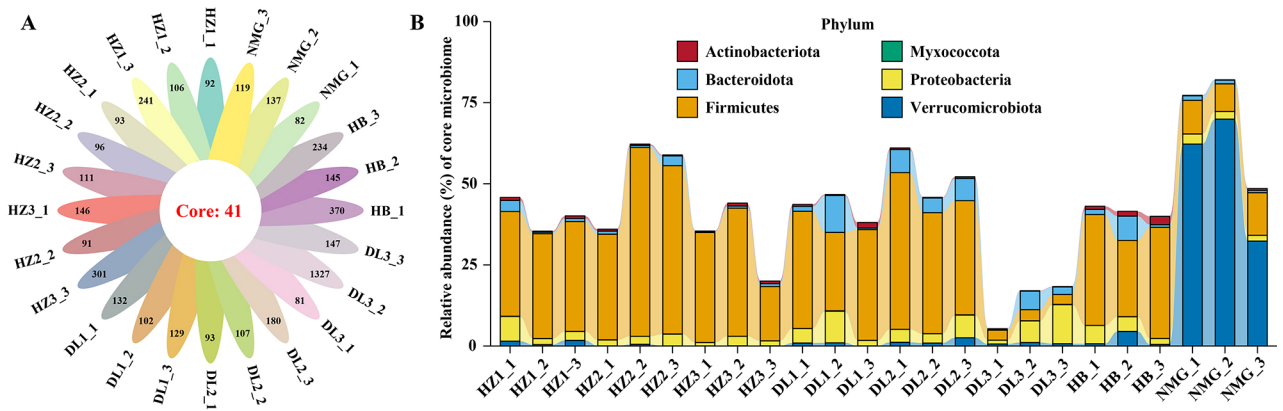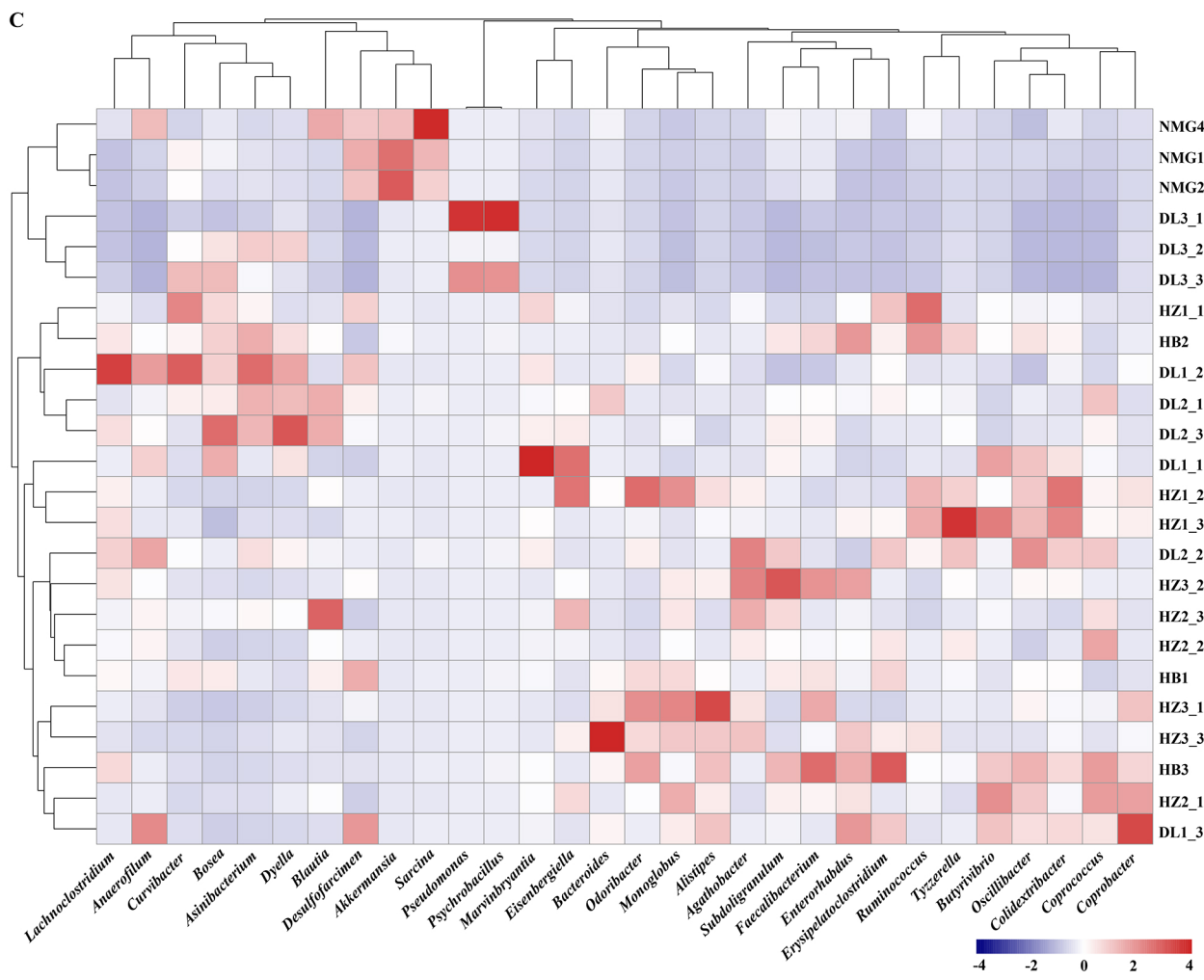

Supplement: Supporting Information 1 — Figure S1: Multidimensional analysis of the fecal microbiota characteristics of great bustards Otis tarda. (A) Distribution of core shared taxa and unique taxa among different samples. (B) Differences in relative abundances at the phylum level among different samples. (C) Heatmap of abundances at the genus level for core taxa among different samples. [file 5587641.f1.pdf]

A

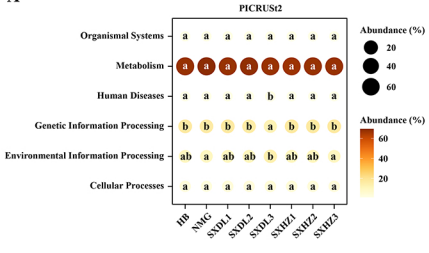

B

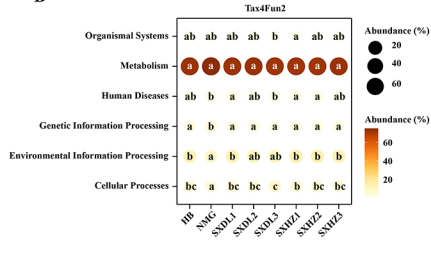

C

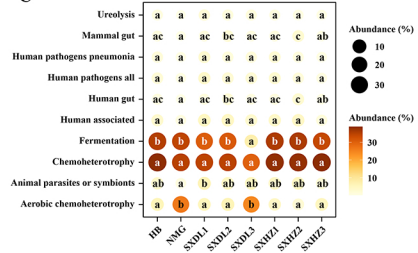

Supplement: Supporting Information 2 — Figure S2: Functional prediction analysis of the great bustard intestinal microbiota based on multiple tools. (A) Distribution of functional categories by PICRUSt2. (B) Distribution of functional categories by Tax4Fun2. (C) Subfunctional categories and ecological associations by FAPROTAX. [file 5587641.f2.pdf]
